# Methylation-dependent *SOX9* expression mediates invasion in human melanoma cells and is a negative prognostic factor in advanced melanoma

**DOI:** 10.1186/s13059-015-0594-4

**Published:** 2015-02-22

**Authors:** Phil F Cheng, Olga Shakhova, Daniel S Widmer, Ossia M Eichhoff, Daniel Zingg, Sandra C Frommel, Benedetta Belloni, Marieke IG Raaijmakers, Simone M Goldinger, Raffaella Santoro, Silvio Hemmi, Lukas Sommer, Reinhard Dummer, Mitchell P Levesque

**Affiliations:** Department of Dermatology, Faculty of Medicine, University Hospital Zürich, and University of Zürich, Wagistrasse 14, CH-8952 Zürich, Switzerland; Faculty of Mathematics and Natural Sciences, Institute of Molecular Life Sciences, University of Zürich, Zürich, Switzerland; Cell and Developmental Biology, Institute of Anatomy, University of Zürich, Zürich, Switzerland; Institute of Veterinary Biochemistry and Molecular Biology, University of Zürich, Zürich, Switzerland

## Abstract

**Background:**

Melanoma is the most fatal skin cancer displaying a high degree of molecular heterogeneity. Phenotype switching is a mechanism that contributes to melanoma heterogeneity by altering transcription profiles for the transition between states of proliferation/differentiation and invasion/stemness. As phenotype switching is reversible, epigenetic mechanisms, like DNA methylation, could contribute to the changes in gene expression.

**Results:**

Integrative analysis of methylation and gene expression datasets of five proliferative and five invasion melanoma cell cultures reveal two distinct clusters. *SOX9* is methylated and lowly expressed in the highly proliferative group. *SOX9* overexpression results in decreased proliferation but increased invasion *in vitro*. In a B16 mouse model, sox9 overexpression increases the number of lung metastases. Transcriptional analysis of *SOX9*-overexpressing melanoma cells reveals enrichment in epithelial to mesenchymal transition (EMT) pathways. Survival analysis of The Cancer Genome Atlas melanoma dataset shows that metastatic patients with high expression levels of *SOX9* have significantly worse survival rates. Additional survival analysis on the targets of *SOX9* reveals that most SOX9 downregulated genes have survival benefit for metastatic patients.

**Conclusions:**

Our genome-wide DNA methylation and gene expression study of 10 early passage melanoma cell cultures reveals two phenotypically distinct groups. One of the genes regulated by DNA methylation between the two groups is *SOX9. SOX9* induces melanoma cell invasion and metastasis and decreases patient survival. A number of genes downregulated by *SOX9* have a negative impact on patient survival. In conclusion, *SOX9* is an important gene involved in melanoma invasion and negatively impacts melanoma patient survival.

**Electronic supplementary material:**

The online version of this article (doi:10.1186/s13059-015-0594-4) contains supplementary material, which is available to authorized users.

## Background

Melanoma is an aggressive skin cancer that originates from melanocytes, that is, pigment cells that reside in the basal layer of the epidermis and are derived from the neural crest during early development [1]. It is the most life-threatening neoplasm of the skin and is considered a major health problem due to rising incidence and mortality rates [2,3]. Melanoma is a tumor with a high degree of heterogeneity and this phenotypic heterogeneity is reversible [4-7]. In addition to being a challenge for basic research, melanoma plasticity is a major hurdle for successful treatment [8]. Investigating the molecular basis of phenotypic heterogeneity is crucial to better understand melanoma progression and should provide useful insights for the development of more effective therapies.

In an effort to elucidate the molecular mechanisms of melanoma progression, significant differences have been detected between melanoma cells from the same lesion [4,6,9]. We and others have found that melanoma cells generally express two distinct gene expression signatures, that these signatures correlate with *in vitro* characteristics and these phenotypes are reversible depending on their cellular microenvironments [10-12]. One signature is characterized by the upregulation of several melanocytic genes like *MITF*, *TYR*, and *DCT*. These melanoma cells are highly proliferative and weakly invasive *in vitro* and so are named the proliferative phenotype. The other signature is characterized by the upregulation of many mesenchymal genes such as *WNT5A*, *TGF*β, and *FGF2*. In contrast to the proliferative cells, these cells are highly invasive but have a low proliferative capacity *in vitro* and are thus named the invasive phenotype. Meta-analysis of all available melanoma microarray datasets available on the NCBI GEO database confirmed these two gene signatures in 86% of the 536 melanomas [13]. Immunohistochemical analyses of MITF and WNT5A, markers of the proliferative and invasive phenotype, respectively, of human primary and metastatic melanomas displayed an anti-correlative staining pattern confirming that these phenotypes exist *in vivo* [14]. Together these findings culminated in the phenotype switching model for melanoma progression, in which melanoma cells respond to changing micro-environmental signals, such as hypoxia, by reprogramming their gene expression patterns to switch between states of proliferation and invasion [9,15]. Thus, phenotype switching has important implications in melanoma progression. Invasive phenotype cells characterized by low MITF expression, have stem-like properties [16], including the ability to initiate tumors with high efficiency [17]. Consequently, tumors comprise a mix of MITF positive and negative melanoma cells [18].

DNA methylation provides a stable and heritable gene regulatory mechanism for which melanoma cells could alter the expression of many genes [19]. Aberrant DNA methylation is a mechanism known to cause tumorigenesis [20]. Tumor suppressor genes become silenced by hypermethylation of their promoter region, thus promoting tumorigenesis. Global hypomethylation has been observed in many cancers, including melanoma, to decrease with progression of the disease [21-23]. DNMT3a and DNMT3b, the *de novo* DNA methyltransferases, were shown to have increased expression in metastatic melanomas compared to primary melanomas [24]. Another group showed that DNMT3a is required for melanoma development and metastasis in a melanoma mouse model [25]. Several signaling pathways have been shown to be deregulated as a result of aberrant DNMT-dependent methylation in melanoma, which include MAPK, WNT, PI3K, pRB, and pathways in cell cycle, apoptosis, invasion, and metastasis [26]. Progressive global DNA hypomethylation has been observed in malignant melanocyte transformation, and surprisingly transformation was blocked in the presence of 5-Aza-2-deoxycytidine (decitabine, Aza), a DNMT inhibitor [22]. It would suggest that targeted hypomethylation is required for malignant transformation and not overall global hypomethylation caused by Aza treatment. This is supported by our observation that treating proliferative melanoma cells with Aza had no measureable effect on their invasive abilities (data not shown). 5-Aza-2-deoxycytidine treatment of various melanoma cell lines was shown to increase SOX9 expression and induce expression of p27 and p21 [27]. SOX9 is a transcription factor involved in neural crest specification [28] and SOX9 overexpression in melanoma cell lines have been shown to induce cell cycle arrest in a p21 dependent manner [29]. Taken together, it would suggest that DNA methylation has a crucial role in malignant transformation and progression by altering the landscape of the methylome to promote tumor progression, and SOX9 is one of the targets of DNA methylation that induces cell cycle arrest.

In this study, we examine the expression of the DNMTs between the proliferative and invasive melanoma cell cultures and describe the differential melanoma methylome by MeDIP-chip. We confirm that SOX9 expression is regulated by DNA methylation and has a role in cell cycle regulation, invasion *in vitro* and *in vivo* and could be a prognostic marker for overall survival in metastatic melanoma patients.

## Results

### Proliferative melanoma cells have higher levels of global DNA-methylation

**Figure 1:**
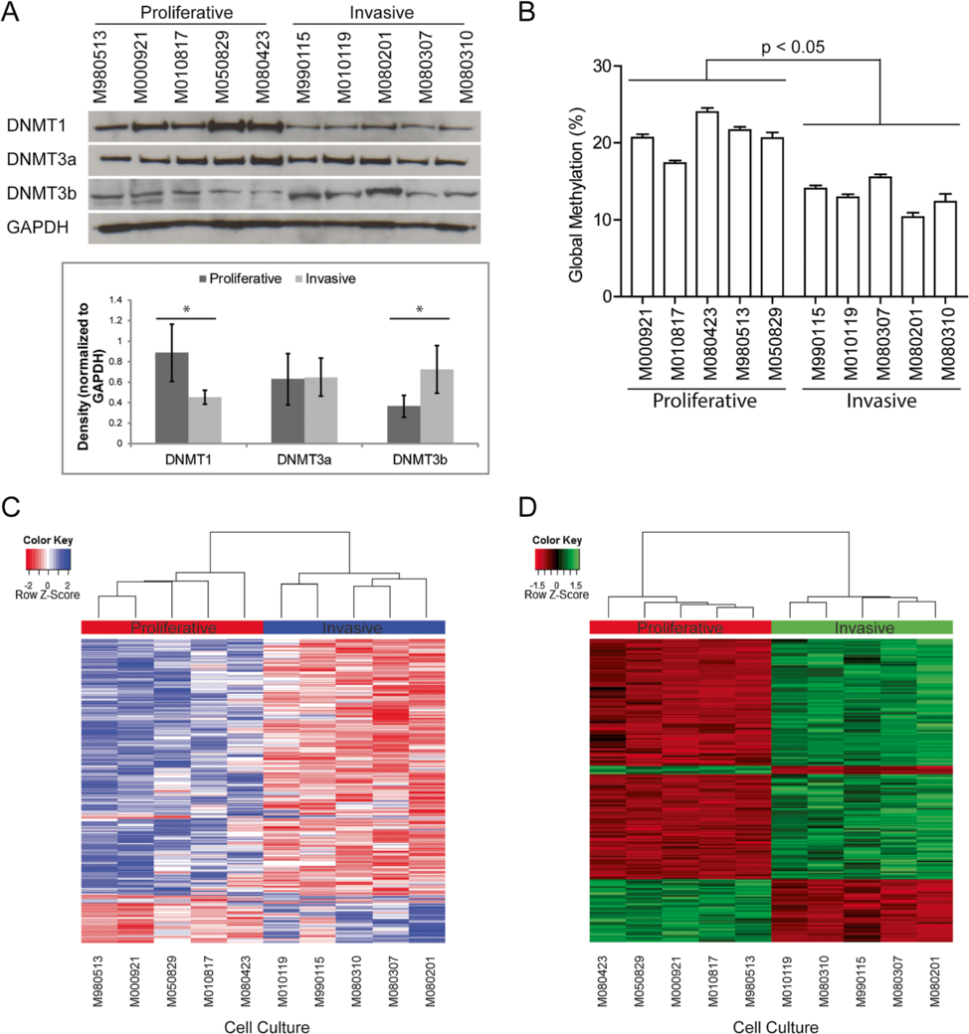
**Phenotype specific expression of DNMTs and DNA methylation patterns. (A)** Western blot for DNMT1 and DNMT3a and DNMT3b on five proliferative phenotype and five invasive phenotype cell lysates. GAPDH was used as a loading control. Optical density of each band was measured and normalized to GAPDH intensity. **(B)** Global methylation analysis by ELISA for methyl-cytosine on five proliferative cell cultures and five invasive cell cultures. There was a significant difference (*P* <0.05, Student’s t-test) in global methylation between the proliferative and invasive phenotype melanoma cells. **(C)** Heat map representing the 406 gene promoters differentially methylated between the proliferative and invasive phenotype cultures. **(D)** Heat map representing the top 250 genes differentially expressed between the proliferative and invasive phenotype.

We have previously established melanocytic markers like MLANA to distinguish between the proliferative and invasive phenotype on a cohort of primary melanoma cell cultures (Additional file 1: Figure S1) [11,30]. To investigate if methylation differences exist between the proliferative and invasive melanoma phenotypes, we compared five proliferative (M000921, M010817, M080423, M980513, and M050829) and five invasive (M990115, M010119, M080201, M080307, and M080310) melanoma cell cultures for expression of *de novo* DNA methyltransferases DNMT1, 3a, and 3b (Figure 1A). We observed that the invasive phenotype melanoma cell cultures had about 51.1% less expression of DNMT1 as compared to the proliferative phenotype melanoma cell cultures (Figure 1A). DNMT3b had about 50% less protein expression in the proliferative melanoma cell cultures compared to the invasive melanoma cell cultures (Figure 1A). However, DNMT3a was not differentially expressed between the proliferative and invasive phenotype (Figure 1A). Global methylation analysis by methyl-cytosine ELISA showed that the invasive phenotype cells have significantly less DNA methylation in their genome compared to the proliferative phenotype cells, 13.0% to 20.9%, respectively (Figure 1B). This raises the possibility that differential methylation exists between the proliferative and invasive phenotype.

### A 73-gene signature is significantly differentially methylated and expressed in proliferative melanoma cells

The difference in global methylation levels and protein expression of DNMT1 and DNMT3b prompted us to investigate the methylation profiles of the proliferative and invasive phenotype melanoma cells. To determine which CpG islands were differentially methylated between the proliferative and invasive phenotypes, we immunoprecipitated methylated DNA from five proliferative and five invasive melanoma cell cultures by MeDIP [31] followed by hybridization to Nimblegen Human DNA Methylation 3x720K CpG Island Plus RefSeq Promoter Arrays. This array contains 720,000 probes for 22,532 promoter regions and 27,728 CpG islands. We calculated the differential methylation levels between the five proliferative and five invasive melanoma cells with a sliding window ANOVA test with the R package DMR supplied from Nimblegen. We found 406 gene promoters to be significantly and differentially methylated between the proliferative and invasive phenotypes (Figure 1C). A total of 320 promoter regions were hypermethylated in the proliferative phenotype and 86 promoter regions were hypermethylated in the invasive phenotype. The greater number of hypermethylated regions in the proliferative phenotype would be consistent with the global methylation data.

Gene expression data for the 10 melanoma cell cultures previously generated by us [13] were reanalyzed for differential gene expression between the proliferative and invasive phenotypes using the R package limma [32]. A total of 1,750 genes were differentially expressed between the proliferative and invasive phenotype (fold change >2, FDR corrected *P* <0.05) (Figure 1D). We then analyzed the relationship between the promoter methylation status and mRNA expression levels for all genes in both datasets. Genes were filtered for a peak score >2 for methylation, fold change >2 for gene expression and an FDR-corrected *P* value <0.05. A total of 73 genes showed both significant differential DNA methylation and significant differential expression between the proliferative and invasive phenotype (Additional file 2: Table S1). Sixty-two genes from the proliferative phenotype had hypermethylated promoters and low RNA expression and 11 genes in the invasive phenotype had hypermethylated promoters and low RNA expression as compared to the proliferative phenotype. This suggests that methylation has a role in regulating a portion of the genes differentially expressed between the proliferative and invasive phenotype. We hypothesized that the 73 genes with both differential DNA methylation and mRNA expression between the proliferative and invasive melanoma cells were likely to be true targets of epigenetic regulation in melanoma. To determine which groups of genes were functionally important, we performed pathway analysis of the 73 genes on MetaCore. We looked for enrichment of pathways under GO processes, process networks, and Pathway maps (Additional file 3: Table S2). Interestingly, we observed significant enrichment in pathways involved in EMT, melanoma, and cell differentiation. We decided to focus on *SOX9* for validation due to its known function in melanocyte differentiation and melanoma progression [29,33,34].

### Sox9 expression is silenced in proliferative melanoma cells through promoter DNA methylation

**Figure 2:**
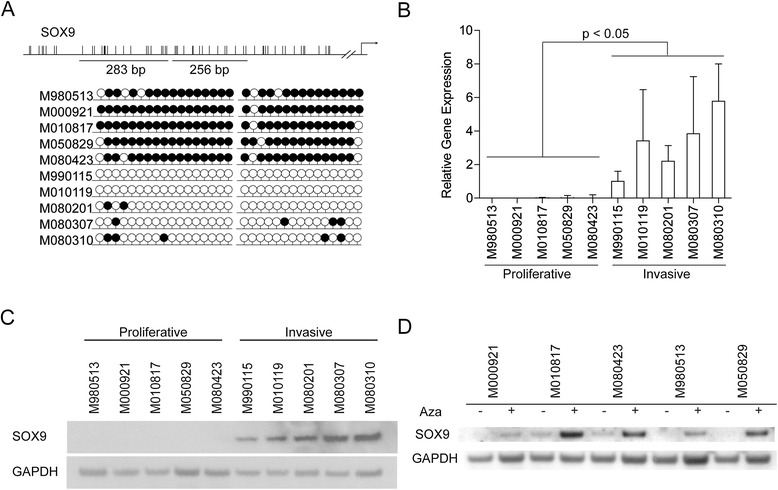
**Validation of SOX9 methylation. (A)** Lollipop diagrams of bisulphite sequencing of SOX9 promoter. Black lollipops are methylated CpGs; white lollipops are unmethylated CpGs. A minimum of five clones were sequenced for each cell culture. **(B)** mRNA expression of SOX9 normalized to the housekeeping gene RPL28 across 10 melanoma cell cultures. Results are presented as mean +/− s.d., n = 3. Statistical significance of differential expression between the proliferative and invasive phenotype cell cultures was determined by Student’s t-test. **(C)** Western blot for SOX9 in 10 melanoma cell cultures. GAPDH served as loading control. **(D)** Western blot for SOX9 of five proliferative melanoma cell cultures treated with 5-aza-2-deoxycytidine (Aza).

From the methylation array, the area that had the most enrichment for methylation was about 2 kb upstream of the *SOX9* transcriptional start site thus we validated the CpG island located there via sequencing of bisulfite-treated genomic DNA in the 10 melanoma cell cultures (Figure 2A). There are three predicted transcription factor binding sites in that upstream promoter region of *SOX9* for *MEF2*, *E2F*, and *HNF3B*. We analyzed the DNA methylation status of a cluster of 17 CpGs across a 283-bp region and 15 CpGs across a 256-bp region of a CpG island, located approximately −2,500 bp to −2,000 bp upstream of the *SOX9* transcriptional start site. The majority of CpGs in both regions of the *SOX9* promoter were consistently methylated in the proliferative phenotype melanoma cell cultures and consistently unmethylated in the invasive phenotype melanoma cell cultures.

To confirm that promoter DNA hypermethylation correlated with transcriptional silencing of SOX9, we assessed mRNA levels using real-time RT-PCR in the 10 melanoma cell cultures. *SOX9* mRNA was expressed robustly in the invasive phenotype melanoma cell cultures compared to the proliferative phenotype melanoma cell cultures (*P* <0.05) (Figure 2B). Protein expression of *SOX9* was detected in all invasive phenotype melanoma cell lysates, but little to no expression of *SOX9* was seen in the proliferative phenotype melanoma cell lysates (Figure 2C). To validate that *SOX9* is indeed regulated by DNA methylation, we treated the five proliferative phenotype melanoma cell cultures with 5 μM 5-Aza-2′-deoxycytidine (a DNMT inhibitor) for 72 h. Re-expression of *SOX9* was detected by western blot (Figure 2D). Thus, *SOX9* expression is regulated by DNA methylation between the proliferative and invasive phenotype.

### SOX9 mediates proliferation and invasion in melanoma cell cultures

**Figure 3:**
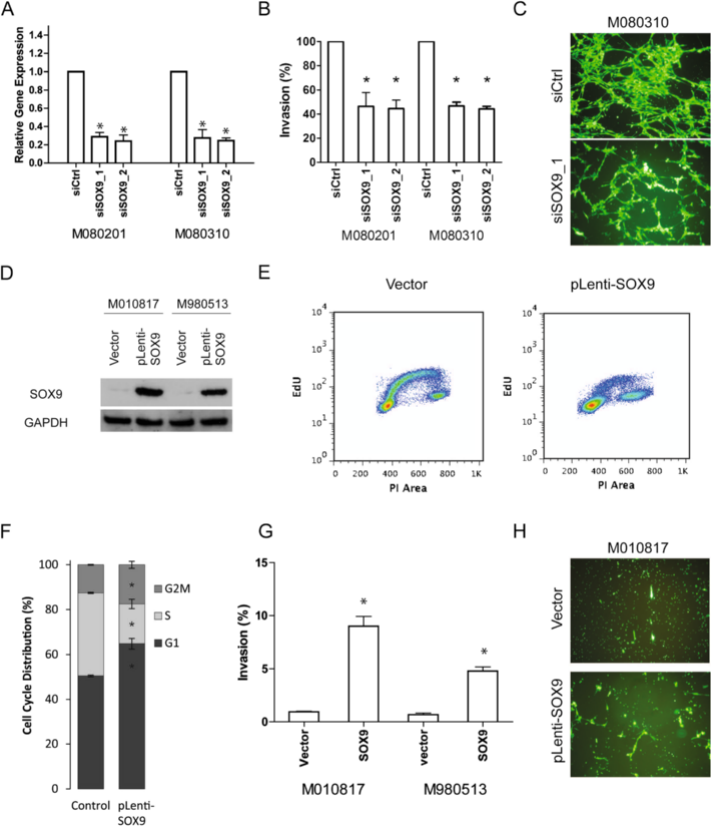
**SOX9 mediates invasion and cell cycle arrest. (A)** SOX9 knockdown by siRNA in two melanoma cell cultures M080201 and M080310. **(B)** Boyden chamber assay for siSOX9 knockdown in M080201 and M080310. **(C)** Representative picture of the Boyden chamber assay for knockdown of SOX9 in M080310. Top panel shows M080310 cells treated with control siRNA (siCtrl). Bottom panel shows M080310 cells treated with siSOX9_1. **(D)** SOX9 overexpression by lentiviral expression in proliferative phenotype melanoma cell cultures M010817 and M980513. **(E)** Proliferation assay by EdU pulse in cell cultures transfected with empty vector and pLenti-SOX9. **(F)** Cell cycle analysis by PI staining for cell cultures transfected with empty vector and pLenti-SOX9 (top and bottom left panels). Quantitation of cell cycle analysis (* = *P* <0.05, right panel). **(G)** Boyden chamber assay for SOX9 overexpressing melanoma cell cultures M010817 and M980513 (* = *P* <0.05). **(H)** Representative picture of the Boyden chamber assay for SOX9 overexpression in M010817. Top panel shows M010817 transfected with empty vector. Bottom panel shows M010817 transfected with SOX9.

*SOX9* is expressed in the invasive phenotype and we have previously described the greater invasive potential of invasive versus proliferative phenotype melanoma cells [30,35]. We hypothesized that some of the differentially expressed genes could have a role in generating this invasive capacity, thus we wanted to see if *SOX9* would have a role in invasion. SOX9 was knocked down with siRNA, and then the invasive ability of two invasive phenotype melanoma cell cultures was measured: M080201 and M080310. Treatment with two independent siRNAs for *SOX9* achieved about 70% knockdown of *SOX9* mRNA in M080201 and M080310 (Figure 3A). The invasive capacity of M080201 and M080310 decreased significantly (*P* <0.05) from 30% to 11.5% and from 64.8% to 36.6%, respectively, after 48 h treatment with siRNA targeting *SOX9* (Figure 3B and C). Proliferation was unaltered from *SOX9* knockdown (data not shown).

Consistent with this observation, we overexpressed *SOX9* in proliferative phenotype melanoma cell cultures (that is, M010817 and M980513) by lentiviral transfection (Figure 3D). Overexpression of *SOX9* was previously shown to drive melanoma cells into cell cycle arrest [29]. We measured proliferation and cell cycle progression by EdU and PI staining, respectively, and observed the cells transfected with vector have 50.5% in G1 phase, 37% in S phase, and 12.5% in G2/M phase, whereas the cells overexpressing SOX9 have 64.8% in G1 phase, 17.7% in S phase, and 17.5% in G2/M phase (*P* <0.05) (Figure 3E and F). The invasive capacity of M010817 and M980513 were significantly increased from 0.95% to 9.0% (*P* <0.05) and from 0.67% to 4.8% (*P* <0.05) from *SOX9* overexpression (Figure 3G and H). In concordance with previously published data on *SOX9* in melanoma, we also see G1/G0 arrest when SOX9 is overexpressed along with increased invasion.

### SOX9 induces a partial invasive phenotype in proliferative melanoma cells

**Figure 4:**
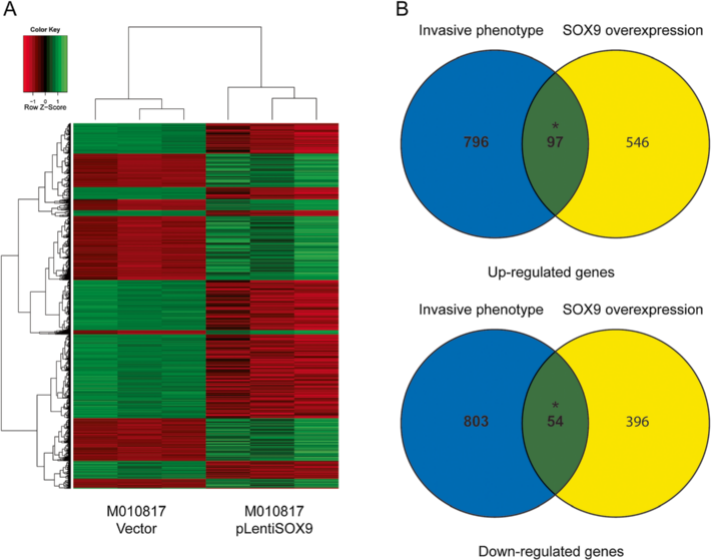
**Microarray analysis of SOX9 overexpression. (A)** Heat map of M010817 cells overexpressing SOX9. **(B)** Overlap of upregulated genes and downregulated genes between the SOX9 microarray and 10 melanoma cell culture microarray.

To determine the effect of *SOX9* overexpression on the proliferative phenotype, we performed microarray analysis of M010817 cells overexpressing *SOX9*. We detected 643 genes downregulated at least two-fold and 450 genes upregulated at least two-fold (*P* <0.05 (Figure 4A, Additional file 4: Table S3). We overlapped the gene signature from the SOX9 overexpression microarray with the gene signature from the 10 melanoma cell culture microarray to ask if *SOX9* induced genes are enriched in the invasive phenotype. There were 98 genes that were upregulated and 55 genes that were downregulated in both the *SOX9* overexpression and invasive phenotype gene sets. Hypergeometric distribution of the overlap of the *SOX9* microarray with 10 melanoma cell culture array was significant (*P* <0.001) (Figure 4B). Thus, *SOX9* apparently regulates about 10% of the genes that define the invasive phenotype gene set. This suggests that *SOX9* activation contributes to the invasive phenotype but other factors are also required for the full transition.

### *In vivo* function of SOX9 overexpression

**Figure 5:**
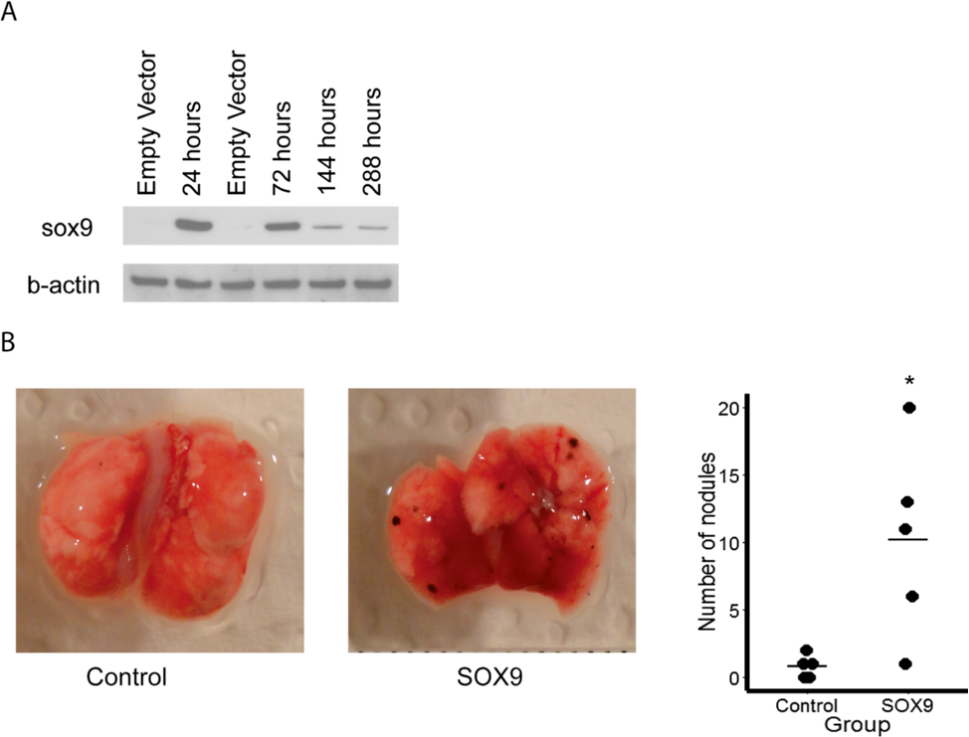
***In vivo*** **function of sox9 overexpression. (A)** Western blot analysis of transient sox9 overexpression in B16F1 up to 12 days. **(B)** Representative pictures of B16F1 cells intravenously injected into C57BL/6 J mice 12 days after injection (n = 5 mice per group). Quantification (right graph). * = *P* <0.05 Mann–Whitney U test.

To examine the effects of SOX9 *in vivo*, we utilized the B16F1 mouse melanoma cells which do not express sox9 and are known not to metastasis in a tail vein injection assay. We transfected murine sox9 transiently into the B16F1 cells and monitored its expression over 288 h. Expression of sox9 decreases over time but protein is still detectable at 288 h (Figure 5A). To assess the *in vivo* metastatic potential of sox9, C57BL/6 J mice were intravenously injected with B16F1 cells transfected with sox9 and empty vector (Figure 5B). Twelve days after injection, the mice were sacrificed and the lungs were analyzed for tumor nodules. B16F1 cells expressing sox9 had significantly more metastases compared to control, *P* <0.05.

### Validation with TCGA melanoma dataset

**Figure 6:**
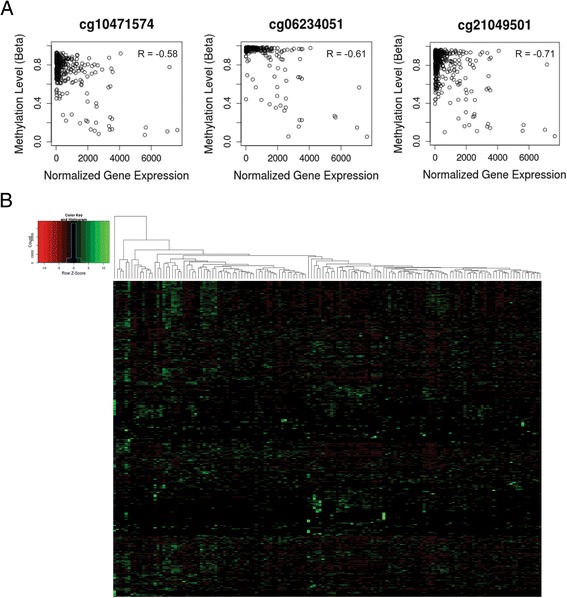
**Validation of SOX9 methylation in TCGA and correlation with clinical features. (A)** Correlation plots of RNAseq reads to b-values of methylation for SOX9. Three methylation probes of SOX9 are shown here to have significant anti-correlation of gene expression and DNA methylation. **(B)** Heatmap of the 427 genes differentially expressed between the SOX9 high and SOX9 low metastatic melanoma samples.

The Cancer Genome Atlas (TCGA) has a melanoma dataset available for public access which contains over 300 tissue samples with RNAseq, DNA methylation, and clinical data. To validate our claim that *SOX9* is regulated by DNA methylation, we performed a correlation analysis of *SOX9* expression to *SOX9* promoter methylation using the data from the TCGA database. Three consecutive probes cg10471574, cg21049501, and cg06234051 in the *SOX9* promoter region have an anti-correlative association with the expression of *SOX9*, r = −0.58, −0.61, and −0.71 respectively (Figure 6A). Since high DNA methylation of *SOX9* is correlated with low *SOX9* expression it provides strong evidence that DNA methylation regulates *SOX9* expression *in vivo*.

The melanoma dataset contains 68 primary samples and 268 metastatic samples. The 268 metastatic samples are comprised with 172 lymph node tumors, 59 regional cutaneous or subcutaneous metastases, and 37 distant metastases. Due to the diversity of this dataset we analyzed the primary and metastatic samples individually. We segregated the population into thirds by *SOX9* expression. We compared the upper and lower thirds for our analysis labelling them *SOX9* high and *SOX9* low. In both primary and metastatic datasets, the *SOX9* high group had at least three times more expression than the *SOX9* low group. We interrogated clinically relevant factors such as TNM staging, age, gender, and tumor type between the *SOX9* high and *SOX9* low group in the primary and metastatic datasets (Table 1, Additional file 5: Table S4). All parameters were statistically insignificant as tested by the Chi-squared test and t-test for age in the primary melanoma dataset. Only two clinical parameters were significant in the metastatic dataset. T1 was significant 2 vs. 11 (*P* = 0.013) in the *SOX9* low versus *SOX9* high, respectively. N0 was also significant 26 vs. 44 (*P* = 0.031) in the *SOX9* low versus *SOX9* high, respectively.

**Table 1:** **Clinical parameters of metastatic melanoma patients**

**Clinical parameter**	**SOX9 low**	**SOX9 high**	**p value**	
T0	15	13	0.706	
T1	2	11	0.013	*
T2	17	17	1	
T3	20	13	0.223	
T4	16	16	1	
N0	26	44	0.031	*
N1	16	9	0.162	
N2	15	9	0.221	
N3	12	9	0.513	
NX	1	0	1	
M0	67	64	0.793	
M1	2	6	0.157	
Female	29	25	0.586	
Male	45	49	0.68	
Lymph node	48	49	0.919	
Regional cutaneous or subcutaneous metastasis	17	14	0.59	
Distant metastasis	9	11	0.655	
Age	57	59.9	0.213^a^	

TNM stage, gender, and tumor location between the SOX9 high and SOX9 low patients were evaluated by the Chi-squared test.

^a^Age was evaluated by Student’s t-test.

*P <0.05.

**Figure 7:**
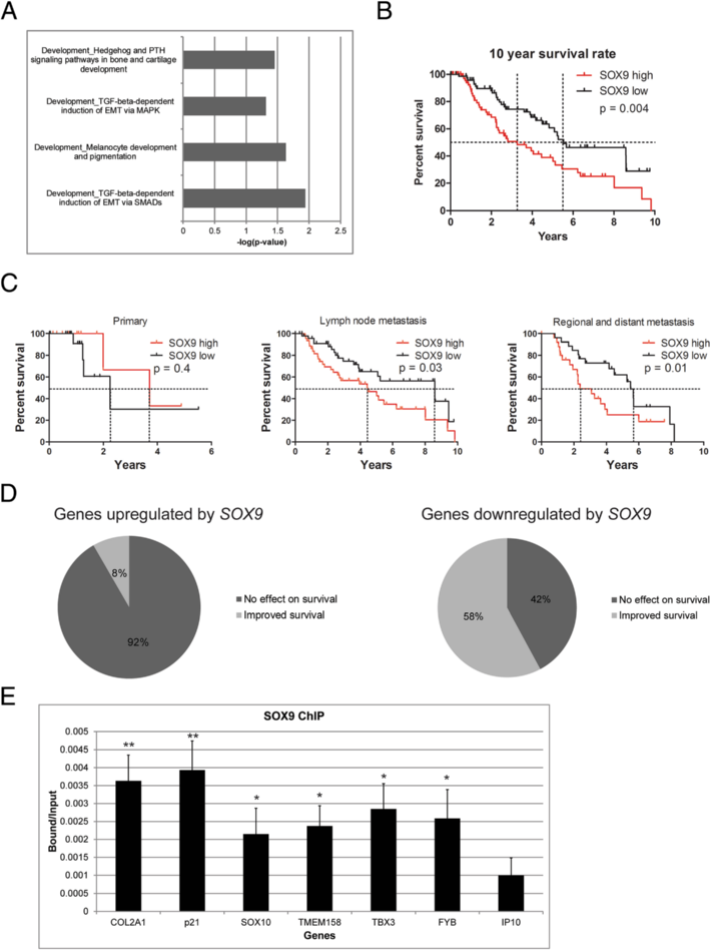
**TCGA analysis of SOX9. (A)** Pathway analysis from MetaCore for SOX9 reveals many pathways involved in EMT. **(B)** Median survival time for patients with high SOX9 expression is 3.9 years (n = 74). Median survival time for patients with low SOX9 expression is 5.8 years (n = 74). The difference in survival is significant (*P* <0.05). **(C)** Survival analysis for high and low SOX9 expression segregated into primary, lymph node, and metastatic cohorts. **(D)** Pie charts displaying the number of SOX9 target genes that have a contribution to patient survival. **(E)** ChIP analysis for SOX9 binding targets. Enrichment of the promoter regions for TMEM158, TBX3, and FYB were similar to positive controls COL2A1, p21, and SOX10 and greater than negative control IP10. Data are shown as bound vs. input. Error bars indicate standard error of the mean of three independent experiments. IgG controls not shown on graph because they were below detection limit.

We next tested if the genes differentially expressed between the *SOX9* high and *SOX9* low patients in the primary and metastatic datasets were the same in our *SOX9* overexpression microarray. A total of 21 genes were differentially expressed between the *SOX9* high and *SOX9* low groups in the primary dataset with a minimum fold change of 2 and FDR corrected *P* value <0.05 (Additional file 6: Table S5). No genes from this set overlapped with the SOX9 microarray. A total of 427 genes were differentially expressed between the *SOX9* high and *SOX9* low groups in the metastatic dataset with a minimum fold change of 2 and FDR corrected *P* value <0.05 (Figure 6B, Additional file 7: Table S6). A total of 31 genes overlapped with the SOX9 microarray. Although the overlap was small, hypergeometric distribution of this overlap was significant (*P* <0.05). To examine the pathways in which, *SOX9* might play a role *in vivo*, we performed pathway analysis on the 427 genes. We saw significant enrichment of many EMT pathway processes such as ‘Regulation of epithelial-to-mesenchymal transition (EMT)’, ‘TGF-beta dependent induction of EMT via SMADs’, and ‘Melanocyte development and pigmentation’ (Figure 7A).

We interrogated if *SOX9* has a role in overall patient survival in all patients regardless of primary or metastatic disease. There was a significant difference in 10-year survival rates between the *SOX9* high and *SOX9* low groups. *SOX9* high patients had a median survival rate of 3.9 years whereas the *SOX9* low patients had a median survival time of 5.8 years (*P* <0.05) (Figure 7B). Cox multivariate analysis was carried out to identify if age, gender, TNM stage, and tumor type were significant contributing factors for 10-year survival of *SOX9* high and *SOX9* low patients. *SOX9* expression (hazard ratio 2.343; 95% confidence interval (CI) 1.402-3.915; *P* = 0.001; SOX9 high vs. SOX9 low) and T4 stage (hazard ratio 2.145; 95% CI 1.01-4.557; *P* = 0.047; T4 vs. T0) were significant (Table 2). We also segregated the patients into primary, lymph node, metastasis and regional/distant metastasis and reassessed survival based on SOX9 expression. We saw that SOX9 expression in primary tumors had no effect on patient survival. However, the cohort of patients with high SOX9 expression in lymph nodes had significantly lower survival (*P* = 0.03), as in the regional/distant metastasis cohort (*P* = 0.01) (Figure 7C).

**Table 2:** **Multivariate cox regression on metastatic melanoma patients**

**Covariate**	**HR**	**Lower 95%**	**Upper 95%**	**p-value**
SOX9 (SOX9 low = 0, SOX9 high =1)	2.343	1.402	3.915	0.001***
Age	0.995	0.977	1.103	0.59
Gender (female = 0, male =1)	0.756	0.457	1.25	0.276
T0 (used as reference)				
T1	0.431	0.117	1.598	0.208
T2	0.943	0.416	2.138	0.888
T3	1.022	0.489	2.132	0.955
T4	2.145	1.01	4.557	0.047*
N0 (used as reference)				
N1	1.673	0.813	3.446	0.162
N2	1.365	0.673	2.771	0.388
N3	1.322	0.574	3.044	0.512
M0 (used as reference)				
M1	2.023	0.672	6.09	0.21
Lymph Node (used as reference)				
Regional Cutaneous or Subcutaneous Metastasis	0.844	0.452	1.577	0.595
Distant Metastasis	1.83	0.957	3.499	0.068

SOX9 expression, age, gender, TNM stage, and tumor location were evaluated in a multivariate cox regression model. Hazard ratio (HR) and the 95% CIs are displayed for each covariate.

*P <0.05.

***P <0.001.

We tested all 31 overlapping genes for survival benefit in the metastatic dataset (Additional file 8: Table S7). Twelve of the genes of this set were upregulated and 19 were downregulated when SOX9 expression was high. Most of the genes, 92% (11/12), that were upregulated when SOX9 expression is high had no influence on patient survival, only one gene 7% (1/12) was associated with improved survival (Figure 7D). This suggests that the genes upregulated by SOX9 are not direct factors for patient survival. Interestingly, 58% (11/19) of the genes downregulated when SOX9 expression is high were associated with improved survival and the other 42% (8/19) had no influence on patient survival (Figure 7D). This suggests that SOX9 represses a group of genes important for patient survival. Taken together, high SOX9 expression leads to poor survival possibly due to the downregulation of several genes that influence patient survival.

### SOX9 binds to the promoter regions of its target genes

Using the SOX9 binding motif to screen for potential binding sites on the promoter regions from the 31 overlapping targets of the SOX9 microarray and the TCGA melanoma dataset, we found 19 of the 31 genes had a potential SOX9 binding site within a 3 kb region upstream from the TSS. To determine whether SOX9 directly binds to the promoter regions of these genes, we performed ChIP analysis using SOX9 antibodies on M010817-SOX9 cells and measured SOX9 occupancy at promoter regions of TMEM158, TBX3, and FYB, for which we could design specific primers for qPCR (Figure 7E). The specificity of this assay was demonstrated by the enrichment of three known SOX9 target sequences, COL2A1 intron 1 [36], p21 [29], and SOX10 [37]), as compared to a non-target gene (IP10). We observed a specific association of SOX9 with TMEM158, TBX3, and FYB, suggesting that TMEM158, TBX3, and FYB are direct targets of SOX9 in melanoma.

## Discussion

Phenotypic, genetic, and epigenetic heterogeneity is a common feature in human melanomas [4,5,14,38]. Tumor subpopulations can be transient and have been seen to switch between phenotypic states *in vivo* [4,6,9,12,39]. We have previously described two subpopulations in melanoma, the proliferative phenotype and the invasive phenotype, which are defined by specific gene signatures, *in vitro* characteristics, and response to drug treatment [11,13,30,35,40]. Briefly, proliferative phenotype melanoma cells are distinguished by a high proliferative capacity and low invasive capacity and the invasive phenotype melanoma cells are distinguished by a low proliferative capacity and high invasive capacity. In this study, we found specific DNA methylation signatures for the proliferative and invasive melanoma phenotypes. We observed the invasive phenotype melanoma cell cultures had modest decrease of 5% in global methylation compared to the proliferative phenotype melanoma cell cultures. This may be due to decreased DNMT1 protein expression in the invasive phenotype melanoma cells. Global methylation levels have been observed to decrease as a cancerous lesion progresses from a benign tumor to metastasis [22,41], and we observed our invasive phenotype melanoma cell cultures had decreased DNA methylation levels and were more invasive than the proliferative phenotype melanoma cell cultures, suggesting the invasive cell cultures have progressed further in malignancy. Differential expression of the *de novo* DNA methyltransferase DNMT3b was also seen between the proliferative and invasive phenotype. These data are consistent with a model in which DNMT1 and DNMT3b have phenotype specificity and contribute to transcriptional heterogeneity by altering the methylation landscape of a melanoma cell in the context of melanoma phenotype switching. Pathway analysis of the 73 gene signature from the DNA methylation and gene expression array lead to the discovery of many transcription networks involved in development. These transcription factors were found to be hypomethylated and highly expressed in the invasive phenotype, which would suggest the invasive melanoma cell cultures may revert to a dedifferentiated state.

A number of other studies that have looked at genome-wide DNA methylation in melanoma have indicated that several tumor suppressors are silenced by DNA methylation compared to normal melanocytes [42-44] and compared to benign nevi [45]. Also, a recent study investigating 5-hydroxymethylation (5-hmC) in melanoma found a global decrease of 5-hmC was necessary for melanoma formation [46]. The results from these studies indicate aberrant DNA methylation is an important process in melanoma development and progression. In our work, we looked at the differences in DNA methylation landscape between 10 primary melanoma cell cultures and uncovered two distinct populations, as previously demonstrated by gene expression microarray analysis from our group [13]. Surprisingly, the targets we found to be differentially methylated between the two phenotypes do not overlap with the targets found to be differentially methylated between normal melanocytes and melanoma, and benign nevi and melanoma. We did not detect any differential methylation in validated methylation gene sets such as *COL1A2*, *NPM2*, *HSPB6*, *DDIT4L*, and *MTIG* from Koga *et al.* [43] or *UCHL1*, *COL1A2*, *THBS1*, and *TNFRSF10D* from Bonazzi *et al.* [42]. As those studies were comparing the methylation state of normal melanocytes to melanoma and in this study we compare within melanoma phenotypes, this might indicate that a different set of pathways are activated or silenced by DNA methylation in melanoma progression compared to melanoma initiation. In either case, it is clear that epigenetic modifications such as DNA methylation play an important role in melanoma initiation as well as progression, and embryonic developmental program reactivation may be one of the critical outcomes of this modulatory activity.

In our study, a subset of our melanoma cell cultures had lower SOX9 expression due to a hypermethylated promoter and the other subset with high SOX9 expression had a hypomethylated promoter. We confirmed that SOX9 is regulated by DNA methylation by treating low SOX9 expressing cells with 5-aza-2-deoxycytidine treatment and saw re-expression of SOX9. To determine if the regulation of SOX9 by DNA methylation is a common mechanism in melanoma or just seen within our melanoma cell cultures, we interrogated the melanoma TCGA dataset for SOX9 and found that SOX9 gene expression and DNA methylation are anti-correlated at three consecutive methylation probes in 293 samples. This provides strong evidence that specific DNA methylation is the molecular mechanism that regulates SOX9 expression in melanoma. Alcazar *et al.* demonstrated that after decitabine treatment of A375 and B16 melanoma cells, the promoter of SOX9 becomes hypomethylated and SOX9 is re-expressed with induction of p27 and p21 for cell cycle arrest [27]. Passeron *et al.* also observed that SOX9 was downregulated in some melanoma cell lines and induction of SOX9 expression in these melanoma cell lines resulted in lower proliferation due to upregulation of p21 [29]. We also overexpressed SOX9 in low SOX9 melanoma cell cultures and observed G1/S cell arrest, which is consistent with the study from Passeron *et al*. Although the proliferation rate is reduced, the invasive capacity of these SOX9 overexpressing cells is increased, which phenocopies the endogenous SOX9 expressing cells. Conversely, knockdown of SOX9 in the invasive phenotype melanoma cells reduced the invasive capacity of the cells. Microarray analysis of SOX9 overexpression revealed an EMT-like transcriptional signature and had 10% overlap with invasive phenotype gene signature which supports the notion that SOX9 is a factor that contributes to the invasive phenotype. *In vivo*, sox9 expression in B16F1 cells increases their metastatic potential causing more tumor lung nodules in the tail vein injection assay. Taken together, SOX9 is a gene that is regulated by DNA methylation and functionally, SOX9 mediates cell cycle progression, invasion, and metastasis in melanoma.

TCGA is a great resource for clinical and next-generation sequencing data on human tumors. We took advantage of the melanoma dataset and demonstrated that *SOX9* expression levels have a significant impact on survival of metastatic melanoma patients but *SOX9* did not have a significant impact on survival in patients with primary melanomas. This could suggest that *SOX9* is required for progression of primary melanoma into metastasis and metastatic tumors with high SOX9 are more aggressive to the patient. There were no clinical metrics that could distinguish SOX9 high or low in primary melanoma. Only T1 and N0 stage in metastatic melanomas were significant between SOX9 high and low. Survival analysis of metastatic patients with high SOX9 expression versus low SOX9 expression revealed a significant difference in the overall 10-year survival rates. Patients with high-SOX9 expressing tumors had a 2.3 times increased risk of death compared to patients with low SOX9 expressing tumors. Based on these findings and the invasive properties of high SOX9 expressing melanomas, it would suggest that SOX9 expression in melanomas could push the tumor toward more aggressive metastasis. Thus, SOX9 could potentially be a prognostic marker for metastatic melanoma.

We performed differential gene expression analysis on the RNAseq dataset where we defined the SOX9 high group as having a minimum of three-fold greater expression than the SOX9 low group. We only saw an overlap of 31 genes between both datasets; however, the overlap was significant as determined by hypergeometric distribution. The contribution of heterogeneity in the melanoma TCGA patient population would be one of the largest factors for the difference in gene signatures between our SOX9 microarray and TCGA RNAseq data. Nonetheless, the significant overlap of genes narrow down the potential targets of SOX9. To confirm that the targets of SOX9 have prognostic value for the patients, we performed survival analysis on all 31 genes. Surprisingly, 58% (11/19) of the genes downregulated by SOX9 were associated with improved survival, which strongly suggests SOX9 represses a set of genes that decrease tumor malignancy. Genes that were upregulated by SOX9 expression had little impact on patient survival which implies that SOX9 expression alone is sufficient to drive disease progression. From this list of 31 genes, 19 of them had a potential SOX9 binding site in its promoter. We could validate TMEM158, TBX3, and FYB as direct targets of SOX9 binding by chromatin immunoprecipitation. FYB is downregulated when SOX9 levels are high suggesting a repressive effect of SOX9 on this gene. FYB is required for inflammatory cytokine production [47] but no known link has been established with melanoma. TMEM158 is upregulated by SOX9 but no clear role has been established for the gene in melanoma. TBX3 is also upregulated by SOX9 and TBX3 is known to cause increased invasiveness in melanoma [48,49], suggesting TBX3 could be an effector gene that drives the invasive phenotype we see in SOX9 high cells and in patients.

## Conclusion

In conclusion, we found SOX9 to be regulated by DNA methylation, and high SOX9 expression leads to poor survival in melanoma patients due to the activation of EMT-like genes and the downregulation of potential tumor suppressor genes in melanoma cells. This was confirmed *in vivo*, and new direct targets of SOX9 that may mediate its function in tumor progression were identified by transcriptional profiling and chromatin-immunoprecipitation. Future therapies targeting SOX9 could be beneficial for patients to prevent progression and especially when combined with therapies targeting cells of the proliferative phenotype. Further investigation would be required to determine if SOX9 would have early prognostic value for tumor malignancy.

## Materials and methods

### Cell culture

Melanoma cell cultures were established from surplus material from primary cutaneous melanoma and melanoma metastases removed by surgery [50]. Written informed consent was approved by the local IRB (EK647 and EK800). Clinical diagnosis was confirmed by histology and immunohistochemistry. Melanoma cells were released from tissue biopsies and grown as previously described [51]. Melanoma cell cultures were maintained in RPMI (Invitrogen, Carlsbad, CA, USA) supplemented with 5 mM glutamine, 1 mM sodium pyruvate, and 10% heat-inactivated fetal calf serum, and cultured at 37°C and 5% CO_2_. As RNA was extract previously from these cell cultures for gene expression array analysis, all cell cultures used for experiments in this paper were within five passages of the RNA isolation time point.

### 5-methylcytosine relative content analyses

Global DNA methylation level was evaluated by MethylFlash Methylated DNA Quantification Kit (Epigentek, Farmingdale, NY, USA) as per manufacturer’s instructions.

### MeDIP assay and analysis

The MeDIP assay was performed as described [31]. A monoclonal antibody to 5-Methylcytidine (BI-MECY-100, Eurogentec, Belgium) was used for immunoprecipitation. The immunoprecipated DNA and sonicated input DNA were differentially labeled with fluorescent dyes (Cy3 and Cy5, respectively) and hybridized to Human DNA Methylation 3x720K CpG Island Plus RefSeq Promoter Arrays (Roche Nimblegen, Madison, WI, USA). Acquisition and analysis was performed using Nimblescan 2.5 and R package DMR provided by Nimblegen. All data have been deposited into NCBI GEO GSE57971.

### Gene expression analysis

Gene expression datasets were obtained from NCBI GEO GSE33728 [13], and analysis was performed by R using the limma package. *P* values were adjusted by FDR multiple hypothesis test correction.

### Bisulphite sequencing

Genomic DNA was extracted from primary melanoma cell cultures and subjected to bisulfite (BS) modification (EZ DNA Methylation Gold Kit, Zymo Research, Irvine, CA, USA). To validate the DNA methylation status of individual DNA molecules, we cloned bisulfite-converted PCR fragments into the pCR2.1 vector using the TOPO-TA cloning kit (Invitrogen, Carlsbad, CA, USA). Individual colonies were screened for the insert, and the region of interest was sequenced using M13 primers. A minimum of five clones were sequenced for each region of interest. Lollipop diagrams were generated using BiQ Analyzer [52]. Primers used for bisulphite PCR are shown in Table 3.

**Table 3:** **Primers for bisulphite sequencing**

**Gene**	**Primer**	**Tm**
SOX9_1	F: 5′-GGATTGGGGTTTTTTATTTTT-3′	59°C
	R: 5′-TTCAATTTTCTTCCCTTTCCT-3′	
SOX9_2	F: 5′-AGGTTATTAGGGTAGATTGGAGG-3′	59°C
	R: 5′5AAATACATATCCCATCACAACC-3′	

### Treatment with decitabine

Decitabine (5-Aza-2′-deoxycytidine, Sigma Chemical (Aza)) was dissolved in DMSO as a 10 mM stock solution, aliquoted, and kept at −20°C. Primary melanoma cell cultures were seeded in Petri dishes (approximately 5,000 cells/cm^2^) in RPMI untreated or treated with Aza (5 μM) for 72 h, with fresh drug-supplemented medium every 24 h.

### mRNA expression analysis

Total RNA was isolated using Trizol according to manufacturer’s instructions (Invitrogen). In total, 1 μg aliquots of RNA were reverse transcribed with Reverse Transcription System (Promega) according to the manufacturer’s instructions. Data collection and analysis were performed by ABI Viia7 Fast Real-Time PCR Systems (Applied Biosystems). Gene expression values of averaged triplicate reactions were normalized to *RPL28* expression levels. RPL28 primers are as follows: 5′-GCAATTGGTTCCGCTACAAC-3′ and 5′-TGTTCTTGCGGATCATGTGT-3′. The primers for RT-PCR were purchased from QIAGEN: SOX9 (Hs_SOX9_1_SG).

### Western blot

Cells were washed twice with cold phosphate-buffered saline (PBS) and lysed at 4°C in lysis buffer containing 20 mM Tris–HCl (pH 7.5), 1% Triton X-100 (Sigma-Aldrich, St Louis, MO, USA), 137 mM NaCl, 10% glycerol, and protease and phosphatase inhibitors (Roche, Basel, Switzerland). Proteins were separated by SDS-PAGE using the NuPAGE SDS-PAGE Gel System (Invitrogen) under reducing conditions. A total of 15 μg of protein was mixed with 9 μL of NuPage LDS sample buffer (4×) (Invitrogen, NP0007), 3.6 μL of NUPAGE Sample Reducing (Invitrogen, NP0009) and filled up to 36 μL with RIPA buffer. This mixture was incubated at 85°C for 10 min while shaking at 900 rpm. Samples were loaded on NuPage precast gels (Invitrogen). Membranes were probed with the following antibodies: SOX9 (GTX109661, GeneTex, Hsinchu City, Taiwan); DNMT1 (ab13537, Abcam, Cambridge, UK); DNMT3a (ab2850, Abcam, Cambridge, UK); DNMT3b (ab16049, Abcam, Cambridge, UK); GAPDH (ab9483, Abcam, Cambridge, UK);

### siRNA knockdown

Silencing RNA (siRNA) transfection of melanoma cells was carried out using INTERFERin transfection solution according to the manufacturer’s protocol (Polyplus-transfection, Illkirch, France). Cells were transfected with 5 nM of siRNA (Qiagen) for 72 h before RNA or protein was extracted. As control siRNA, the All-Star negative siRNA sequence (Qiagen) was used, and gene-specific siRNAs targeting siSOX9 (SI00007595, SI00007609) were obtained from Qiagen.

### SOX9 lentiviral transfection

Lentiviral particles containing plasmids expressing full-length SOX9 cDNA or eGFP were transfected into melanoma cells for 48 h. Media supplemented with 4 ng/mL blasticidin was used for selection. After 1 week of selection, protein lysate was extracted and analyzed for SOX9 expression. Plasmids for eGFP and SOX9 were a kind gift from Dr. Thierry Passeron [29].

### SOX9 transient transfection

Empty vector or vector containing murine sox9 (a kind gift from Prof. Lukas Sommer) was transfected into B16F1 cells with jetPEI (Polyplus, France) according to manufacturer’s protocol. Protein was isolated at 24 h, 72 h, 144 h, and 288 h after transfection and analyzed for sox9 expression by western blot.

### Microarray and pathway analysis

Gene expression of eGFP and SOX9 transfected cells were analyzed using the Affymetrix Human Genome U133 Plus 2.0 Array at the Functional Genomics Center Zurich (FGCZ). Differential gene expression was determined by R package limma [53]. Pathway analysis was performed using MetaCore (GeneGo Inc., New York, USA).

### Proliferation and cell cycle analysis

For cell cycle analysis, the Click-iT EdU Alexa Fluor 647 Flow Cytometry Assay Kit (Invitrogen) was used. Cells were labelled with PI according to the manufacturer’s protocol and the DNA content was measured using a BD FACSCanto II flow cytometer (BD Biosciences) and BD FACSDiva software (BD Biosciences). Cell cycle analysis was performed in triplicate.

### Boyden chamber invasion assay

Cells were seeded on FluoroBlok 24-multiwell Insert System (351157, BD Biosciences) and Biocoat Tumor Invasion System (354165, BD Biosciences). The invasion assay was performed as per manufacturer’s instructions. Migrated and invaded cells were labeled with Calcein AM fluorescent dye (354216, BD Biosciences) and fluorescence was measured with Tecan GENios (Tecan, Männendorf, Switzerland) using 485 nm excitation and 535 nm emission. Relative invasion was calculated as the ratio of the fluorescence of invading cells of the Biocoat Tumor Invasion System divided by the fluorescence of migrating cells of the FluoroBlok 24-multiwell Insert System. Boyden chamber assays were performed in triplicate.

### Viability assay

Cells were seeded in 24-well microplates at a density of 2 × 10^4^ cells, and cell growth was determined with a standard colorimetric assay measuring 3-(4,5-dimethylthiazol-2-yl)-2,5 diphenyltetrazolium bromide (MTT) (Sigma-Aldrich, St Louis, MO, USA) reactivity after 72 h. MTT assays were performed in triplicate.

### Chromatin immunoprecipitation

ChIP analysis was performed as previously described [54]. The Sox9 antibody was from Santa Cruz Biotechnology (sc-20095, Santa Cruz Biotechnology). Primer sequences were designed around SOX9 binding motifs [55,56] from the transcription start site (TSS) to 3 KB upstream of the TSS. Primers for p21, COL2A1, TMEM158, TBX3, FYB, SOX10, and IP10 are shown in Table 4.

**Table 4:** **Primers for ChIP**

**Genes**	**Primer**
COL2A1-F	ATCCTCCTTTGTGAGGCTTGTT
COL2A1-R	AGTACGAGAGAACCCACTGGAC
p21-F	TGATGTGCCACAGTTCACAA
p21-R	TCCTGCCAGTTTTCCTGTTC
TMEM158-F	TCTGCTGTGTTGGAGCCATT
TMEM158-R	GTCTCGCCTTAGTGCTACCG
TBX3-F	CTCGCCCCTTTCTTTCCCTT
TBX3-R	GGGGGTGTTATGAGCCAACA
FYB-F	CTCACATTGCATGGGGACG
FYB-R	ATGGGCTTATCACCGGAAGG
SOX10-F	CCTCTGCCTCGTGTGACTAC
SOX10-R	TCCTGTCTGGAGTGGGCTG-
IP10-F	GGGAAATTCCGTAACTTGGA
IP10-R	AAGCCATTTTCCCTCCCTAA

### *In vivo* metastasis

B16F1 cells were transfected with empty vector or vector containing murine sox9. 2 × 10^5^ cells were injected intravenously into C57BL/6 J mice, five mice per group (Harlan Laboratories). After 12 days mice were sacrificed and lungs were examined for metastasis. Statistics were performed using the Mann–Whitney U test. All animal experiments have been approved by the veterinary authorities of Canton of Zurich, Switzerland, and were performed in accordance with Swiss law.

### TCGA analysis

The SKCM DNA methylation, RNA-seq, and clinical dataset were downloaded on 28 July 2014 for analysis. Normalized reads from the level 3 RNA-seq data were used for analysis. The dataset was segregated into primary tumors and metastatic tumors for analysis. Chi-squared test was performed on the clinical parameters between the SOX9 high and low groups. Differential expression was analyzed with voom from the limma package [53]. Log rank test and Cox proportional hazard ratio were analyzed by the survival R package [57]. DNA methylation β-values were calculated by minfi [58]. Correlation was calculated by Spearman’s rank correlation coefficient.

## Additional files

Additional file 1: Figure S1. MLANA expression between the proliferative and invasive phenotype. Ten melanoma cell cultures were divided into the proliferative and invasive phenotype by expression of MLANA. GAPDH was used as loading control. Additional file 2: Table S1. 73 genes with anti-correlative methylation and gene expression. Analysis of the methylation and expression profiles of the ten melanoma cell cultures produced this list of significant genes with a peak score greater than 2 from the MeDIP methylation array and with a fold change greater than 1 from the gene expression microarray. Additional file 3: Table S2. Pathway analysis of 73 gene signature. Genes were uploaded to Metacore and analyzed for significant pathway enrichment in GO Processes, Process Networks and Pathway Maps. Additional file 4: Table S3. Differential gene analysis of SOX9 overexpression. Limma output table for differentially expressed genes between SOX9 overexpression and control M010817 melanoma cells. Additional file 5: Table S4. Clinical parameters of primary melanoma patients. TNM stage, gender, and tumor location between the SOX9 high and SOX9 low patients were evaluated by the Chi-squared test. Age was evaluated by Student’s t-test. * represents p < 0.05. Additional file 6: Table S5. Differential gene analysis of SOX9 high and low primary melanoma patients. Limma output table for SOX9 high primary melanoma patients versus SOX9 low primary melanoma patients. Additional file 7: Table S6. Differential gene analysis of SOX9 high and low metastatic melanoma patients. Limma output table for SOX9 high metastatic melanoma patients versus SOX9 low metastatic melanoma patients. Additional file 8: Table S7. Overlap of SOX9 overexpression and metastatic melanoma patients. List of genes overlapping from M010817 melanoma cells overexpressing SOX9 and SOX9 high metastatic melanoma patients.
