# Supplementary figures and images for: Methylation-dependent SOX9 expression mediates invasion in human melanoma cells and is a negative prognostic factor in advanced melanoma
Source: Genome Biol. 2015 Feb 22;16(1):42. doi: 10.1186/s13059-015-0594-4 (PMC4378455; doi:10.1186/s13059-015-0594-4)

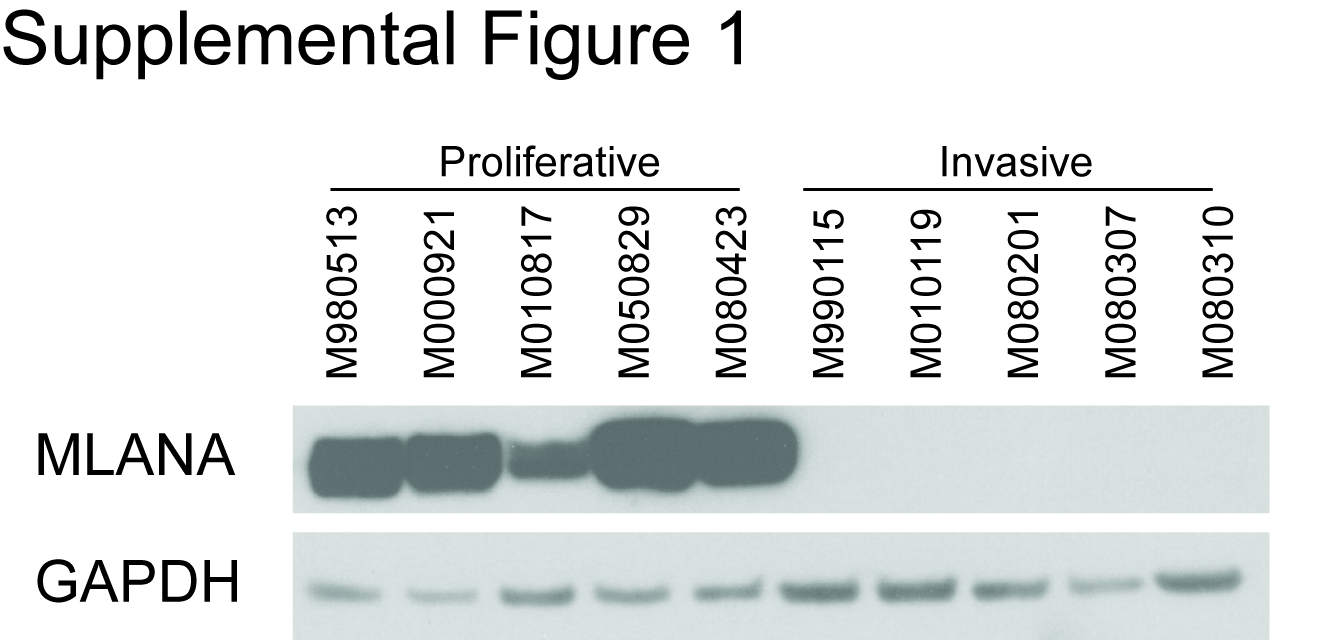

Supplement: Additional file 1: Figure S1. — MLANA expression between the proliferative and invasive phenotype. Ten melanoma cell cultures were divided into the proliferative and invasive phenotype by expression of MLANA. GAPDH was used as loading control. [file 13059_2015_594_MOESM1_ESM.tif]
